# Phase transformation and enhanced blue photoluminescence of zirconium oxide poly-crystalline thin film induced by Ni ion beam irradiation

**DOI:** 10.1038/s41598-021-96961-w

**Published:** 2021-09-03

**Authors:** Vishnu Chauhan, Deepika Gupta, Nikhil Koratkar, Rajesh Kumar

**Affiliations:** 1 University School of Basic and Applied Sciences, Guru Gobind Singh Indraprastha University, New Delhi, 110078 India; 2 Department of Mechanical Engineering and Materials Science, Rensselaer Polytechnic Institute, 110 8th Street, Troy, NY 12180 USA

**Keywords:** Condensed-matter physics, Nanoscale materials

## Abstract

Swift heavy ions (SHI) irradiation of Nickel (Ni) beam with different ions fluence bring the modifications in the functional properties of radio frequency (RF) grown zirconium oxide (ZrO_2_) nanocrystalline thin films. X-ray diffraction analysis affirms the monoclinic to tetragonal phase transformation and diminishing of peak at higher fluence 1 × 10^14^ and 2 × 10^14^ ions/cm^2^ induced by electronic excitation caused by SHI. Zirconium oxide thin films exhibit the same thickness (195 nm) of virgin and irradiated samples and whereas the nanocrystalline thin films have the elemental composition in proper stoichiometry (1:2) as analyzed by rutherford backscattering spectroscopy (RBS). Photoluminescence measurements confirm the blue emission of virgin and irradiated sample recorded at excitation wavelength 270 to 310 nm. The intensity of obtained emission bands varies with fluence which is interpreted in terms of generation and annihilation of defect centers. The characteristic A_g_ and B_g_ Raman modes of monoclinic and tetragonal ZrO_2_ are obtained at different positions. Moreover, the nanocrystalline ZrO_2_ thin films exhibits the most prominent absorption phenomenon in the visible range and the irradiation cause significant decrease in band gap to 3.69 eV compare to the virgin ZrO_2_ sample (3.86 eV). XPS analysis indicates the shifting of the core levels Zr 3d and O 1s towards higher binding energy and spin—orbit splitting of different states. The findings in this research justify that the irradiated thin films can be a potential candidate for designing of new materials, intense radiation environments, nuclear reactors, nuclear waste systems, clean energy sources.

## Introduction

Neutron, proton and high energy ions irradiation can generate large number of defects including defects cluster, bubbles, voids and dislocations in metallic materials. Consequently, ion radiation may cause void swelling and tailoring or modifications in the properties of materials in terms of radiation hardening and irradiation induced defects and creep^1–5^. Modifications induced in ceramic materials by electronic excitations are the key factor for understanding the generation of defects under high energy-heavy ions irradiation impacts. Electronic sputtering caused by electronic excitation is also the direct measurement of atomic displacement near the surface of the material. It has been extensively studied for ceramics and oxide materials and suggested that the modifications in properties of materials are important factors for electronic sputtering which explain the larger change in yield due to larger electronic sputtering^6^. Ion beam irradiation has the advantage of controlled release of specific elements and change in properties of ion-irradiated films as compared with the heat treated films. The microstructures of ceramics produced by different methods have been extensively studied in various reports whereas investigations on ion-irradiated thin films have been limited. In addition, limited reports are available on understanding the effect of high energy ion irradiation on atomic structure and properties of existed phases of high energy ion irradiated zirconium oxide thin films^7^. The interaction of energetic ions with the target material is the deciding key factor of ion beam induced material modifications. Incident ions lose their energy and material gains energy when they pass through the target material. Furthermore, the keen interest in SHI irradiation on metal oxide semiconductor materials is due to the influence of irradiation on physical properties and chemical bonding of the materials. Swift heavy ions (SHI) irradiation is known as a good tool to create a variety of defects which is responsible for formation of structural defects like strain and disorder in materials, responsible for modifying the structural, optical, transport, electrical, chemical, surface morphological and magnetic properties of different materials^8^. There are some important and promising properties of ceramics that are used for various purposes. It is worth mentioning that protective coatings of ceramics are considered for accident tolerant fuel cladding for light water reactors (LWRs)^9^. The fine grain size of coatings provides the benefit from strength and chemical inertness of ceramic materials which withstand with the favorable high radiation tolerance demonstration in nanomaterials^9–11^. Zirconium oxide finds its extensive use as ceramic material because it has important applications in scientific and engineering fields such as electronic devices, electrolyte because its surface acidity and basicity, protective and thermal barrier coatings, biomedical engineering, solid-oxide fuel and oxygen sensors^12–17^. Unlike AlTiO, zirconium oxide is simple binary oxide that can be easily deposited in thin films forms using various techniques such as chemical vapor deposition (CVD), atomic layer deposition (ALD), plasma spraying techniques^18^, plasma coating method^19^, and reactive RF magnetron sputtering technique^20^. It is more chemically stable than Copper (I) oxide Cu_2_O^21^. Several reports are available on swift heavy ion irradiation induced electronic excitation in alkali, earth alkali halides and oxide materials such as SiO_2_, UO_2_, Y_3_Fe_5_O_12_, LiF, Gd_3_Ga_5_O_12_, TiO_2_ and Wo_3_^22–26^. Sharma et al.^27^ studied the structural and microstructure evolution in tin monoxide (SnO) thin films (200 nm) deposited by RF sputtering technique. The investigation was carried out by irradiating SnO thin films under 150 MeV Au ions with the fluence 1 × 10^11^, 1 × 10^12^ and 5 × 10^12^ ions/cm^2^. Among the oxide materials, some data is available for ZrO_2_ with respect to high and low energy ion irradiation^28–33^. Singh et al. reported the evolution of tetragonal phase using micro-Raman studies and crystalline to crystalline phase transition (monoclinic to tetragonal) in ZrO_2_ thin film during 120-MeV Ag swift heavy ions irradiation^56^. The investigations on structural and electronic study confirmed the dissolution of monoclinic phase and monoclinic to tetragonal phase transformation in ZrO_2_ thin films under 200 MeV Ag-ions beam irradiation^34^. Moreover, Lu et al. reported the amorphization of nanocrystalline monoclinic zirconium oxide by 1.69 GeV Au ions irradiation that suggested the ZrO_2_ as highly radiation tolerance material^35^. Similarly, Rathika et al.^36^ investigated the structural, optical and transport properties of WO_3_ thin films under the effect of 200 MeV Ag ion beam irradiation with a range of fluence 5 × 10^11^ to 1 × 10^13^ ions/cm^2^. The motivation of the study includes the structural transition and modifications in featured properties of ZrO_2_ caused by swift heavy ions irradiation. In present work, we report the evolution of modifications induced on nanometer-scale structural, optical, morphological, electronic and chemical features in ZrO_2_ thin films as a function of high energy different ion irradiation dose. Often, thin films are used to analyze the fundamental influence of high energy radiation on nanocrystalline materials and considered as suitable model systems. The obtained findings in irradiated ZrO_2_ thin films are expected to be similar as in case of bulk samples provided that the virgin microstructural features are comparable with the irradiated samples.

## Material and methods

ZrO_2_ thin films were grown on Si and glass substrate by RF sputtering technique at RPI, New York, USA. The details of deposition of pure ZrO_2_ thin films have been reported elsewhere^48^. X-Ray Diffraction (XRD) technique was used to study the structural properties using grazing angle incidence. XRD patterns were recorded with Bruker D8 advance X-ray diffractometer in θ-2θ geometry arrangement with Cu-K_α_ radiation (λ = 1.5416 Å) at Indian Institute of Technology, Roorkee, India. Rutherford backscattering spectrometry was carried out by beam of ^4^He^+^ ion with 2 MeV, on 5SDH-1.7 MV Tandem accelerator at Inter University Accelerator Centre (IUAC) New Delhi. Perkin Elmer LS 55 Fluorescence spectrometer was used to analyze photoluminescence spectra of virgin and irradiated thin films ZrO_2_ at 270–310 nm excitation wavelength at Guru Nanak Dev University, Amritsar, Punjab, India. Raman scattering was carried out in Renishaw In-Via Reflex micro Raman spectrometer operating at 488 nm Ar ion excitation laser operating at 50 mW power, diffraction grating of 2400 lines/mm and Peltier cooled CCD detector. Absorption, band gap and transmittance spectra of the films were investigated using a dual beam Hitachi U3300 spectrophotometer. X-ray photoelectron spectroscopy (XPS) was used to analyze the chemical properties of ZrO_2_ thin films using Omicron ESCA+, Oxford instrument, Germany. The surface morphological study of the samples was carried out by atomic force microscopy technique using Bruker multimode 8 with Nanoscope V in tapping mode.

### Ion beam irradiation

In order to study the induced modifications, thin films were irradiated with high energy heavy ions as an alternate technique of neutron irradiation^37^. For the clear analysis of defects caused by ion irradiation, the appropriate ion irradiation experimental parameters were selected. The high energy of the ions was chosen to provide the irradiation effect beyond the thickness (195 nm) of ZrO_2_ thin films. In a set of annealed samples at 600 °C with the irradiation area of 1 × 1 cm^2^, each sample was irradiated using Ni ions obtained from 15 UD Pelletron accelerator at Inter University Accelerator Center (IUAC), New Delhi, India. The 100 MeV Ni^7+^ ions beam was used for SHI irradiation. Ion Irradiation was carried out in vacuum (10^–6^ Torr) and beam current of high energy ions was sustained at 1 pnA throughout the irradiation. The samples were irradiated at room temperature with a range of fluence 5 × 10^12^, 1 × 10^13^, 1 × 10^14^ and 2 × 10^14^ ions/cm^2^. The ion beam was focused using magnetic scanner to obtain fluence uniformity across the sample area. The fluence values were determined by collecting the charge falling on sample mounted on metal ladder.

### Discussion on the possible mechanisms

**Figure 1:**
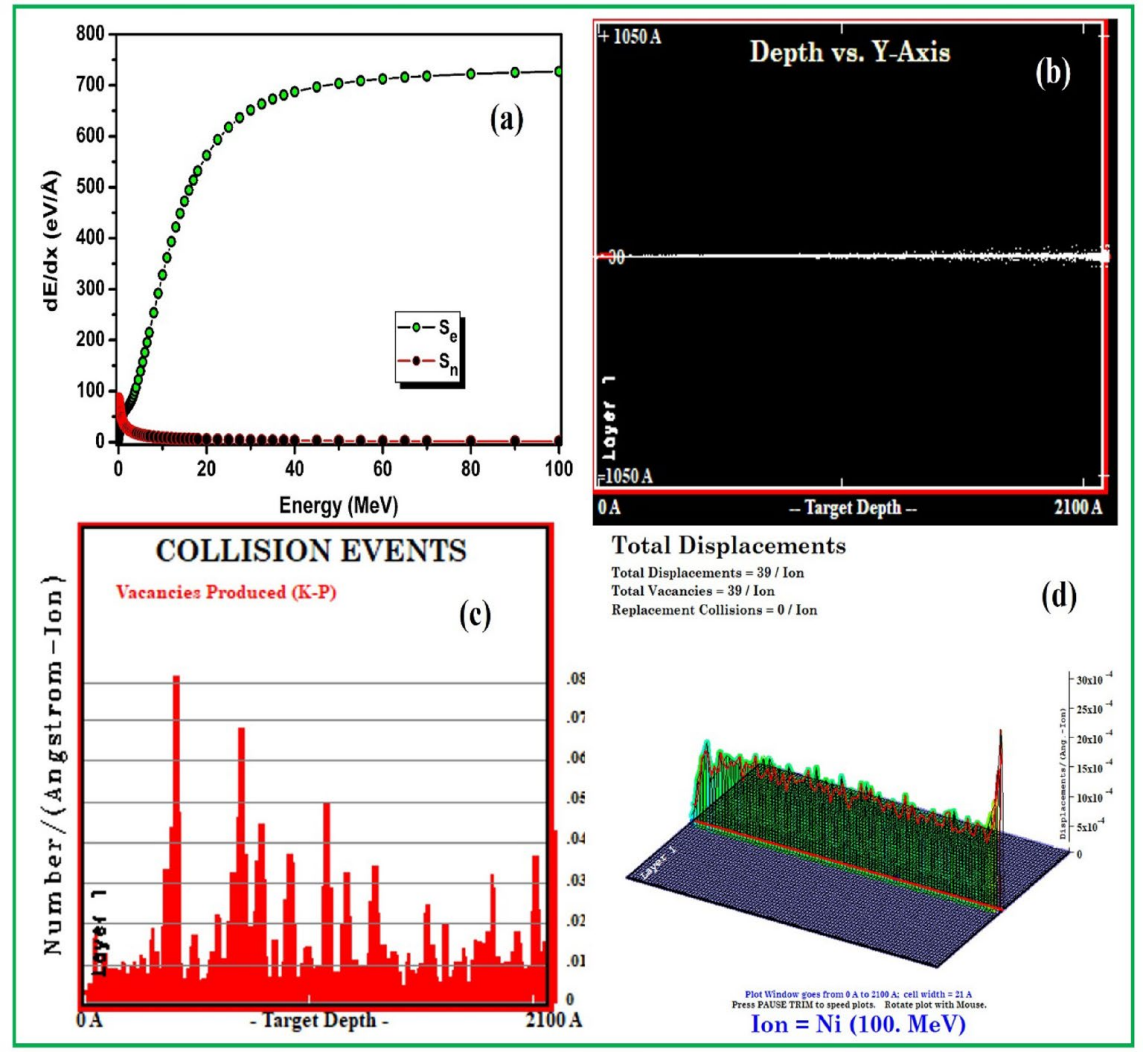
The S_e_ and S_n_ profile (**a**) Ni^7+^ ion beam in ZrO_2_ thin films, detailed calculation with full damage cascade (**b**) depth vs. Y-axis (**c**) collision events and (**d**) Target displacement 3D respectively for Ni ion irradiation.

Swift heavy ion irradiation induced modifications in optical, structural, chemical, morphological and electronic properties are judged by different characterizations that can be understand by studying the energy transfer processes of high energy ions irradiations. The electronic (S_e_) and nuclear (S_n_) energy losses of 100 MeV Ni^7+^ ion irradiation in ZrO_2_ thin films were determined using SRIM software. Figure 1a depicts the energy loss profile of Ni ion beam in ZrO_2_ thin films. The detailed calculation of 100 MeV Ni ions with full damage cascade information have been determined for 5000 ions by using TRIM program as shown in Fig. 1b–d. It is evident from the Fig. 1b that Ni ions lose most of their energy by the process of electronic energy loss. Here, in high energy regime S_e_ (7.273E + 02 eV/Å) are dominant over S_n_ (1.447E + 00 eV/Å). The projected range of 100 MeV Ni ions in ZrO_2_ thin films is 19.77 μm. Thus it is concluded that all ions are passed through the thin films (210 nm) deep into substrate. The observed ion beam irradiation effects have to be understood as consequences of SHIs induced electronic energy transfer. In constructed ways, the focus towards swift heavy ions (SHIs) irradiation is to attain targeted irradiation for the modifications in the properties of materials. The energetic ions transfer their energy to the solid material instantaneously into confined volume. Extremely excited zone quickly “cools” down within few picoseconds due to the dissipation of energy in cold surroundings. Such highly solid state excitations cannot be attained by any other technique for tailoring the properties of the material^38^. In SHIs irradiation, two models (1) Coulomb explosion and (2) thermal spike are accounted for explanation of electronic energy losses^39,60^. The kinetic energy of swift heavy ions is transferred to the targeted electrons within very short time 10^–17^ s upon traversing solid target. The targeted electrons make positively charged inner cylinder after moving away from the ion path. The generated ions repel each other by Coulomb force also called electrostatic force which ensures that their neutralization time is sufficiently long which rely upon electron density and mobility. The hypothesis is based on the assumption that the mechanical strength of irradiated solid materials must be overcome by coulomb repulsive force. Moreover, if the electrostatic energy is greater than the chemical bond energy, the columbic repulsion will lead to track formation followed by effective atomic motion^40–42^. The ion track formation states the melting, sputtering and material resolidification via formation of new bonds which results into material modifications^43^. Thermal spike model is proposed in two steps, considering the irradiated material as electron gas and atomic lattice to describe the track formation in solid materials. In first step, the energy of the ejected electrons is mutually shared with the other electrons results into local thermallization of electron gas in very short time scale (~ 10^–15^ s). Second step follows the transfer of energy by electronic heat conduction as results of electron–lattice interaction results in large increase of temperature. This model also supports the explanations of defects creation and effect of irradiation temperature in solid materials^40,44^.

## Results and discussion

### X-ray diffraction

**Figure 2:**
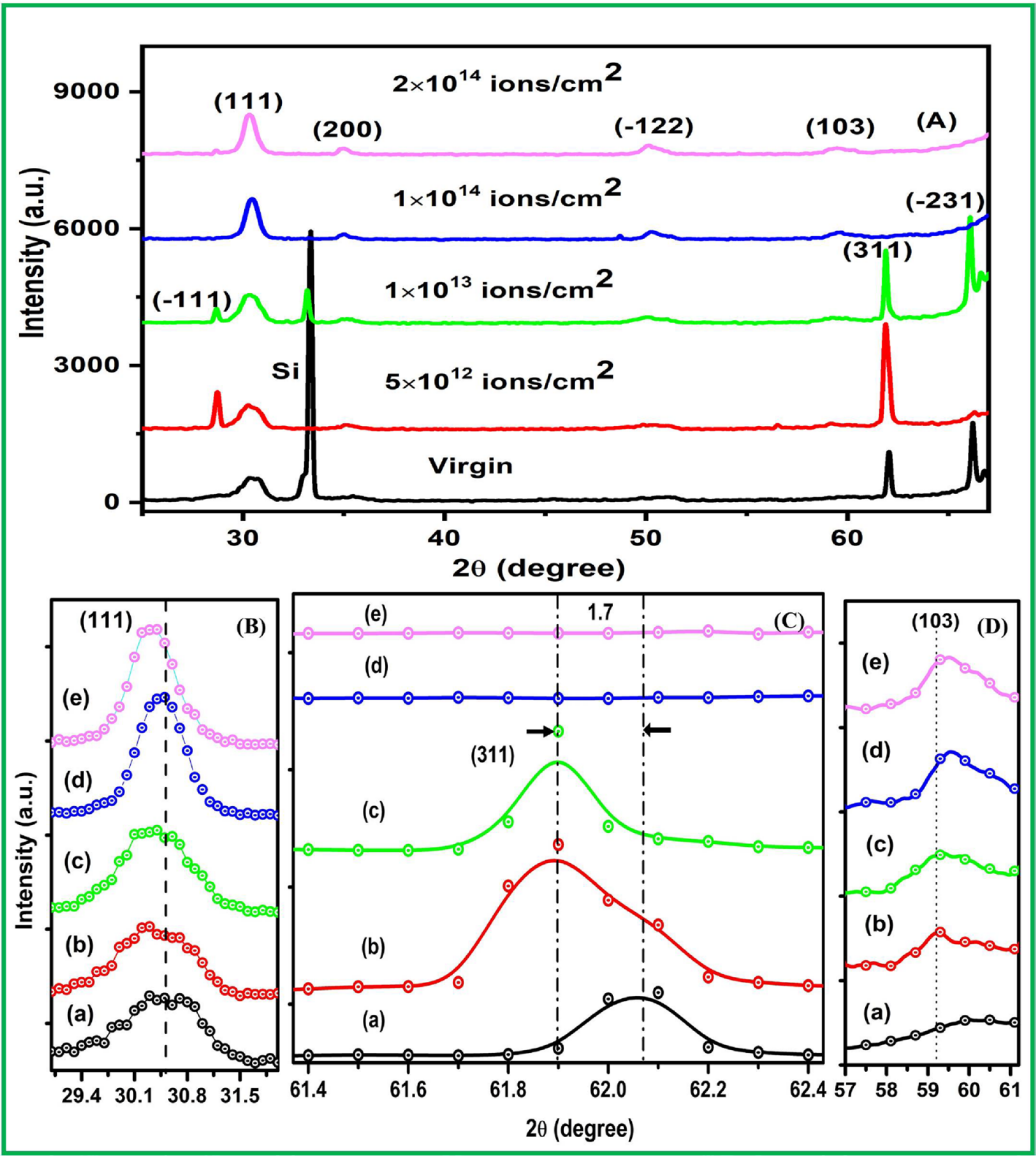
X-ray diffraction pattern (**A**) ZrO_2_ virgin and irradiated samples showing the crystalline nature (monoclinic and tetragonal structure), the magnified image of the reflection planes (**B**) (111) shows peak shifting towards lower angle, also (**C**) plane (311) shows the shifting of peak towards lower angle and (**D**) (103) plane shows the phase transformation (monoclinic to tetragonal) of ZrO_2_ thin films when (a) virgin samples irradiated with Ni ion using fluence (b) 5 × 10^12^ (c) 1 × 10^13^ (d) 1 × 10^14^ and (**e**) 2 × 10^14^ ions/cm^2^.

The XRD patterns of ZrO_2_ virgin and irradiated samples with different ion fluence are shown in Fig. 2A. Moreover, the intense multiple peaks from different planes of thin films indicate that the ZrO_2_ thin films are polycrystalline in nature and exhibit good crystalline quality. From the XRD results, it is observed that there is significant variation in peak intensity. The intensity of the planes (111), (200) and (−122) increased after ion beam irradiation. Increment in intensity is associated with the decrease in density of originated defects through the annihilation process after the ion interaction with the material. This enhancement in intensity of the peak is on account of diffusion of atoms across the boundaries of grains in the target material^45^.

The diffraction peaks of crystal planes (−111), (200) and (311) corresponds to monoclinic structure as compared to standard JCPDs data (# 70-2491). Also, the plane (−122) and (−231) confirm the existence of monoclinic structure (# 37-1487) while the planes (111) and (103) corresponds to tetragonal structure (#JCPDs 17-0923 and 81-1544). The crystallinity of the nanocrystalline ZrO_2_ samples is gradually improved with increase in ions fluence. To determine the crystallite size of virgin and irradiated samples, we have used Scherrer equation for (311) and (111) of monoclinic and tetragonal phase respectively^46^.

1 $$ {\text{D}} = \frac{{K\lambda }}{{\beta \cos (\theta )}} $$

The strain induced in irradiated thin films has been calculated using the given equation

2 $$ \varepsilon  = \frac{{\beta \cos (\theta )}}{4} $$

It is determined that the crystallite size increased and correspondingly strain decreased in virgin and irradiated samples with increasing the ions fluence because of the substantial amount of energy of ions is conveyed to the material and accountable for the evolution of zones with high temperature. Ion irradiation brings about local annealing of the surface of material under the influence of transient heating caused by phonon and electron coupling and is known as thermal spikes. This energy causes the reduction in strain field between the crystallites of irradiated material. Thus, the crystallite size enhanced as function of enhancement in ions fluence. Thus with the ion beam irradiation crystallites quality enhance and this instigates the process of annihilation of defects which is one of the significant reasons for the increment in the crystallite size^47^.

For the peak (111), the magnified image can be seen in Fig. 2B, the apparent shift is observed towards lower angle 2θ after Ni ion beam irradiation which might be due to release of residual stress in the crystal structure. Similarly, magnified image of the peak (311) shows the significant peak shifting by 1.7 (2θ) towards lower angle up to the fluence of 1 × 10^13^ ions/cm^2^, at higher fluence the peak is diminished as shown in Fig. 2C. Universally, it is known that the peak shifting towards lower angle signify the presence of tensile stress while peak shifting towards higher angle signify the compressive stress. The magnified image of the plane (103) is shown in Fig. 2D, and it is observed that virgin sample has no sign of any phase but swift heavy ion beam irradiation results into evolution of new peak. The intensity of this peak is increased with increasing the ions fluence and it confirms that the Ni 100 MeV SHI irradiation cause monoclinic to tetragonal phase transformation of the samples. It is owing to the reason that ion beam irradiation instigate the damage of ZrO_2_ lattice and enhance more vacancies of oxygen, inner stress in materials and defects elongation is accountable for transformation of phase^48^.

At higher fluence after 1 × 10^13^ ions/cm^2^, the peak (311) is vanished which indicates the partial amorphization in the materials as compared to the virgin and lower fluence irradiated samples. For higher dose, the intensity of the plane (111) is as expected as smaller for virgin sample and increased with the ions fluence, whereas the full width half maxima is reduced with ions fluence as shown in Table 1.

**Table 1:** Crystallite size and strain measurements from XRD data of virgin and Ni beam irradiated ZrO_2_ thin films with ions fluence of 5 × 10^12^, 1 × 10^13^, 1 × 10^14^ and 2 × 10^14^ ion/cm^2^.

Sample	Crystal plane (111)				Crystal plane (311)			
	Peak position (2θ)	Β_h k l_ (rad) × 10^–2^	D (nm)	Strain (ε) (× 10^–3^)	Peak position (2θ)	Β_h k l_ (rad) × 10^–3^	D (nm)	Strain (ε) (× 10^–3^)
**Virgin & 100 MeV Ni**^**7+**^** SHI ion irradiated ZrO**_**2**_** thin films**								
Virgin	30.47	2.25	6.67	20.67	61.89	4.01	42.08	1.67
5 × 10^12^	30.40	2.01	7.48	18.48	61.9	5.58	30.25	2.32
1 × 10^13^	30.35	1.88	7.97	17.38	62.05	3.14	53.77	1.30
1 × 10^14^	30.44	1.29	11.64	11.87	–	–	–	–
2 × 10^14^	30.30	1.25	12.29	11.60	–	–	–	–

### Rutherford backscattering spectrometry

**Figure 3:**
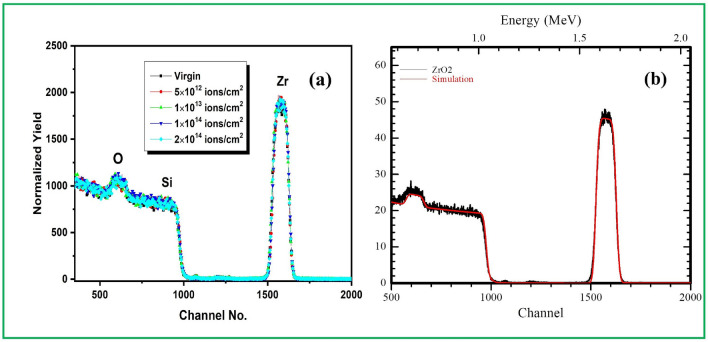
RBS spectra of (**a**) virgin and irradiated with 100 MeV Ni ions at fluence 5 × 10^12^, 1 × 10^13^, 1 × 10^14^ and 2 × 10^14^ ions/cm^2^ of ZrO_2_ samples and (**b**) Simulated Rutherford backscattering spectra of virgin ZrO_2_ thin film.

RBS spectra and atomic concentration for virgin and irradiated samples are presented in Fig. 3a. The rutherford backscattering spectrometry measurements were performed with He^+^ particles with 2 MeV on 5SDH-1.7 MV Tandem accelerator (PARAS). Typical beam constant current and charge used in the RBS analysis were 12 nA and 12 μC respectively. It is seen that the synthesis of the ZrO_2_ thin films confirmed with the peak detected at channel number 1577 and 603 assigned to the zirconium (Zr) and oxygen (O) respectively. The peak width of both Zr and O is similar that support the same thickness of the samples. Nevertheless, the atomic content of Zr as well as O in the thin films remained the same (Zr—0.623, O—0.375) and the thickness (195 nm) of the samples was calculated using simulation program (SIMNRA). Figure 3b shows the simulated RBS spectra of virgin ZrO_2_ thin film.

### Photoluminescence spectroscopy

PL (Photoluminescence) is one of the significant technique for optical analysis of semiconductors. It has capability to detect the existence of excitons fine structure, impurities and defects which generally influence the quality of materials^49^. Based on the synthesis technique, ZrO_2_ with high purity displays the good emission bands.

**Figure 4:**
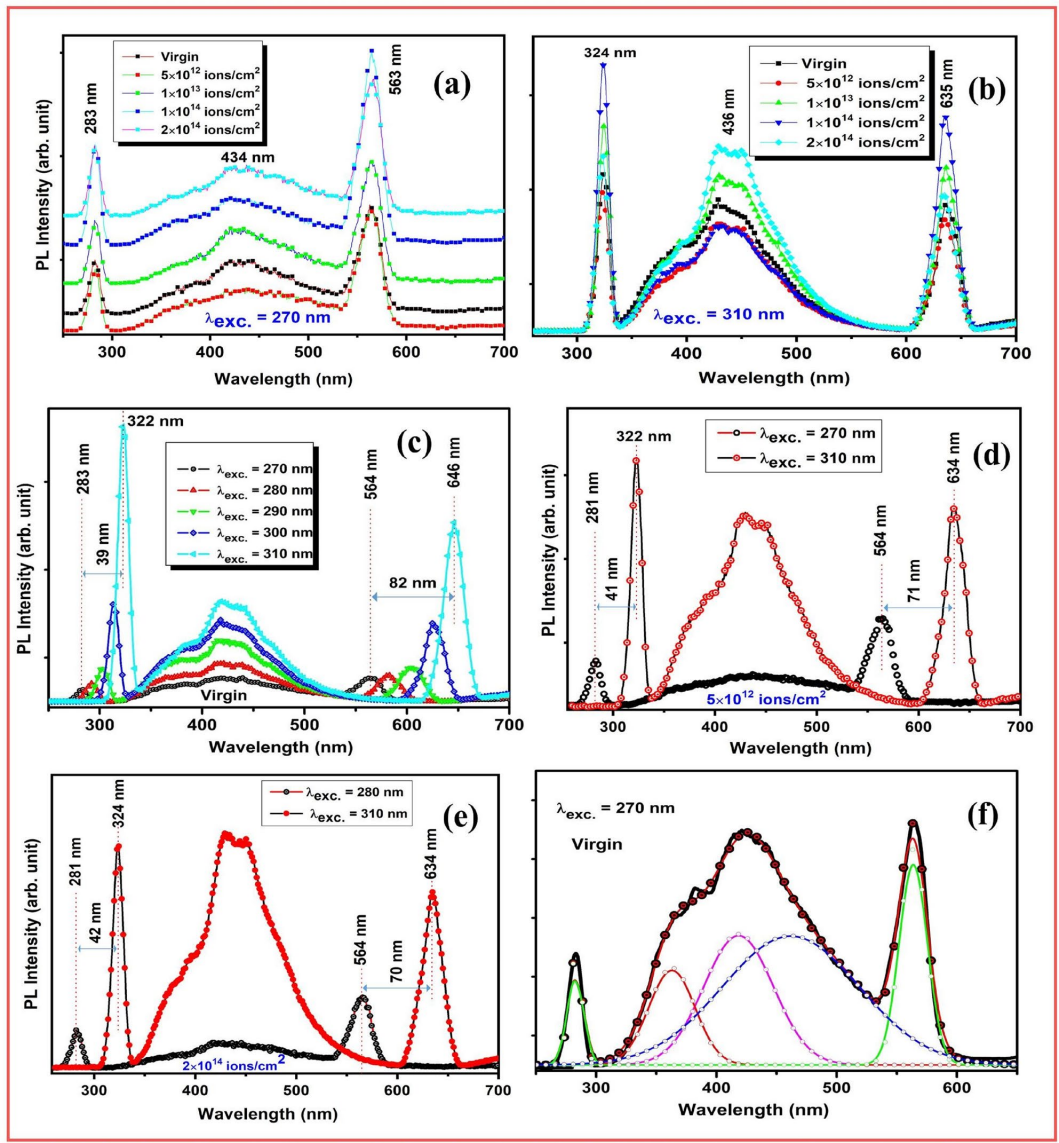
Photoluminescence spectra of (**a**) virgin and Ni ion beam irradiated ZrO_2_ samples excited at 270 nm, (**b**) excited at 310 nm with varying ions fluence, (**c**) PL spectra of the virgin ZrO_2_ thin films indicates the shift in peak films with increasing in excitation wavelength 270 to 310 nm (**d**) PL of Ni^7+^ ion beam irradiated sample at 5 × 10^12^ ions/cm^2^ with excitation wavelength of 270 and 310 nm (**e**) sample irradiated at 2 × 10^14^ ions/cm^2^ with excitation wavelength of 280 and 310 nm and (**f**) De-convoluted PL spectra of virgin sample excited at 270 nm.

PL technique is generally utilized to analyze the crystalline nature and existence of defects in thin films. PL spectra were analyzed for virgin and ion irradiated ZrO_2_ thin films at excitation wavelength of 270 nm wavelength as shown in Fig. 4a. Similarly, Fig. 4b shows the emission spectra of virgin and irradiated samples at excitation wavelength of 310 nm. PL emission band at 450 nm corresponds to blue emission at excitation wavelength of 270 nm wavelength and significant change in the intensity of the peak is not observed with the ion beam irradiation but peak intensity is slightly escalated after 1 × 10^13^ ions/cm^2^_._ The samples irradiated with the different fluence remark that the PL emission bands are additionally intense and the intensity supplemented with the enhancement in ions fluence.

The inferred PL emission band at 434 nm corresponds to broad blue emission may be on account of number of induced defect^50^. The PL intensity is explicitly correlated with the concentration of defects caused by SHI irradiation at range of fluence^51^.

Peak observed at 563 nm corresponds to the yellow emission. Prominently, the intensity of the emission band at 563 nm is increased with escalating the ions fluence. Lokesha et al. reported the PL emission in virgin (un-irradiated) ZrO_2_ samples under swift heavy ions irradiation that confirmed the crystalline virgin sample also exhibit PL emission bands^52^. The origin of defects can be additionally construed as the SHI perforates in the solid material; inelastic collision is anticipated between the target electrons and the ions. Substantially in inelastic collision, it is presumed that ionization and electronic excitation of the target atoms play a significant role in heavy ion irradiation effect on ZrO_2_ thin films. Inelastic collision in SHI lead to transfer of energy which instigates the amorphization/crystallization and appreciable enhancement in defects. Electronic excitation instigated by significant electronic stopping can enervate the oxygen bonds and evolution of oxygen vacancies^53^.

Similar PL emission spectra is observed except the variation in the intensity of the emission bands and shifting of PL peak towards higher fluence when the results are remarked and recorded at excitation wavelength of 310 nm wavelength. Significantly, the intensity of PL bands is enhanced with increasing fluence which signifies the enhancement in defect concentration whereas intensity decreased at maximum fluence 2 × 10^14^ ions/cm^2^.

Figure 4c illustrates the PL spectra recorded for the virgin sample at distinct wavelength from 270 to 310 nm to analyze the variation in emission bands of virgin and irradiated samples. Remarkably, it is observed that the peak obtained at 283 nm when excited with the 270 nm, it is shifted by 39 nm towards higher wavelength when excited at higher wavelength 310 nm. Similar way, the band obtained at 564 nm is shifted towards higher wavelength by 82 nm. Moreover, PL intensity of the emission bands increased with the enhancement in excitation wavelength from 270 to 310 nm. Further, PL spectra recorded at 270 and 310 nm for the sample irradiated with 5 × 10^12^ ions/cm^2^ and again a PL shift is observed by 41 and 71 nm for the intense emission bands as illustrated in Fig. 4d. Moreover in Fig. 4e, the PL emission spectra is shown for higher irradiated sample 2 × 10^14^ ions/cm^2^. In Fig. 4f deconvolution of the virgin sample has been done for the virgin sample excited with 270 nm wavelength. With Implementation of Gaussian deconvolution of the PL spectra, it was noticed that a broad, supplemented and blue emission band is observed at 462 nm with two shoulder bands at 363 and 418 nm which can be associated with the deep level emission instigated by impurities and inherent structural defects in virgin ZrO_2_ thin films like oxygen vacancies and interstitials defects^53^.

### Raman spectroscopy

The each unit cell of monoclinic and tetragonal ZrO_2_ contains four and two molecules per unit cell respectively. Group theory describes that the monoclinic ZrO_2_ contains 36 lattice vibration modes i.e. G_mono_ = 9 A_g_ + 9 A_u_ + 9 B_g_ + 9 B_u_, in which 9 A_g_ and 9 B_g_ are Raman active mode sand 8 A_u_ and 7 B_u_ are IR active modes and rest are the acoustic modes. Whereas, the group theory describes 18 phonon for tetragonal zirconium oxide in which six are Raman active modes i.e. (A_1g_ + 2B_1g_ + 3E_g_). The monoclinic and tetragonal zirconium belongs to ${C}_{2h}^{5}$ (P2_1_/c) and ${C}_{4h}^{15}$ (P4_2_/nmc) respectively^54^. All Raman active modes are strong cation dominant (Zr^4+^) or strong anion dominant (O^2−^). It is well known that the heavy metal Raman modes are mostly on lower band position (below 200 cm^−1^) whereas the light oxygen atoms are dominating towards high band position^55^.

**Figure 5:**
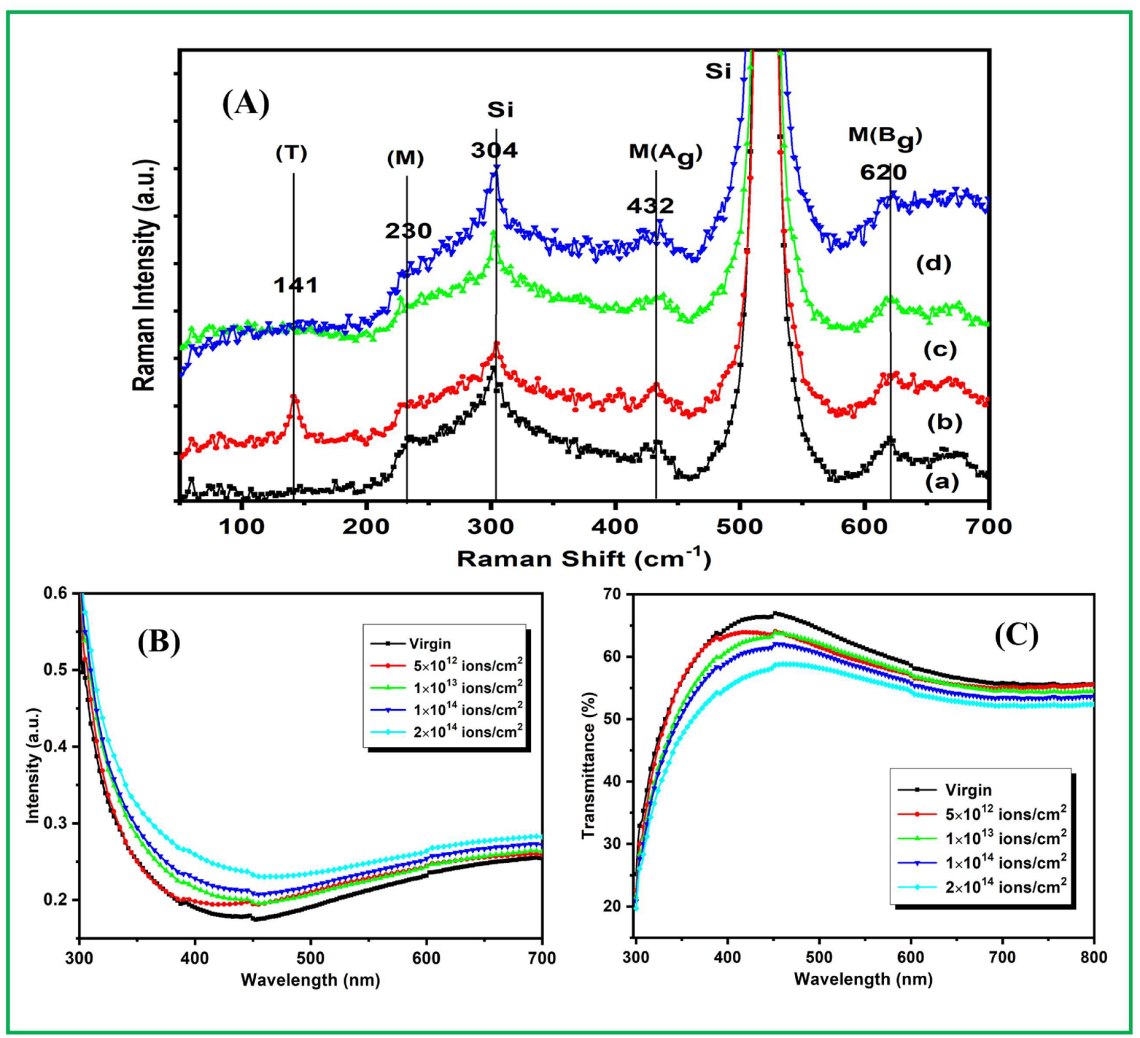
Raman spectra of (**A**) ZrO_2_ thin films irradiated with Ni^7+^ ion beam shows different bands corresponds to monoclinic and tetragonal phase indicating nanocrystalline nature of ZrO_2_ samples, (a) virgin and irradiated samples with the fluence (b) 5 × 10^12^ (c) 1 × 10^14^ (d) 2 × 10^14^ ions/cm^2^, (**B**) Absorption spectra of Ni^7+^ ion beam irradiated ZrO_2_ thin films shows the band edge of ZrO_2_ in all samples and (**C**) Transmittance spectra shows the decrease in band gap and transmittance with increase in ions fluence.

The Raman spectra have been determined at room temperature to analyze the effect of Ni swift heavy ions irradiation on phonon modes of virgin and irradiated ZrO_2_ thin films. Figure 5A shows the Raman spectroscopy signals recorded in ZrO_2_ thin films after and before irradiation with 100 MeV Ni^7+^ ions and processed with the fluence 5 × 10^12^ ions/cm^2^ to 2 × 10^14^ ions/cm^2^ in which five Raman active modes are obtained. Out of five Raman active modes, two characteristic A_g_ and B_g_ modes of monoclinic ZrO_2_ are obtained at 432 cm^−1^ and 620 cm^−1^ respectively^56^. Also, the monoclinic and tetragonal phase identification of ZrO_2_ thin films have been confirmed by XRD results. The high Raman active mode of the Si substrate is obtained at 522 cm^−1^, identical for virgin and irradiated samples^56^. The Raman bands appeared at 141 cm^−1^ (most significant characteristic mode Eg) and 230 cm^−1^ are attributed to tetragonal and monoclinic structure of ZrO_2_ and similar Raman bands are reported by Barberis et al.^57^. Further, the mode observed at 304 cm^−1^ is of Si substrate due to 2TA optical phonon vibration is silicon. The influence of Argon (Ar) ion implantation with 1 × 10^15^ to 1 × 10^17^ ions/cm^2^ ions fluence on the structural properties of ZrO_2_ samples studied by Raman spectroscopy and the shift in Raman band resulted into the stress induced hardening effect and monoclinic → tetragonal (m → t) phase transformation^58^. The Raman active phonon modes located at band position agrees with the reported results^57,58^. Generally, the change in the band width of the modes in virgin and irradiated samples signifies that the modes are affected by the formation of the disorder due to SHI irradiation^5^. The increase in band width of the peaks indicate the increase in disorder, hence the decrease in optical band gap^3^.

No Raman modes are observed for virgin and higher fluence (1 × 10^14^ and 2 × 10^14^ ions/cm^2^) irradiated thin films at band position of 141 cm^−1^. Further, the intensity of the remaining bands decreases with the increasing ions fluence and the similar behaviour is obtained from the XRD peaks. The appearance of Raman active bands show a disordered activation (distortion in ZrO_2_ structure) induced by SHI irradiation^59^. These observations are in accordance with XRD results indicating a controlled phase transformation of ZrO_2_ structure. It is known that ions irradiation may cause residual stress induced and generation of defects within the irradiated layer. In situ micro-Raman investigations have been reported to interpret the experimental data for stiffing of phonons and monoclinic to tetragonal phase transformation in ZrO_2_ thin films under 120 MeV Ag swift heavy ion irradiation^56^.

### Optical study of ZrO_2_ thin films

The optical absorption spectra of virgin and Ni ions irradiated ZrO_2_ thin films examined at room temperature have sharp band edge in ultraviolet region as shown in Fig. 5B. Observed absorption band edge has been changed from 349 to 362 nm that changes accordingly from virgin to Ni ion irradiated samples up to higher fluence 2 × 10^14^ ions/cm^2^. Absorption is increased in visible region with the irradiated ions fluence in thin films. The absorbance spectra contains two parts—first is originated from the direct fundamental optical band gap and other originated from the ion beam irradiation which increase the absorption of the system in visible region. The transmittance in the visible region is found to be between 67 and 58% for virgin and irradiated samples. It is clearly observed that the ions dose (fluence) of Ni ions ZrO_2_ decreases the transparency. The overall 9% decrease in transparency is observed for irradiated thin films as shown in Fig. 5C that could be due to generation of higher concentration of defects in samples cause of high energy irradiation^64^.

**Figure 6:**
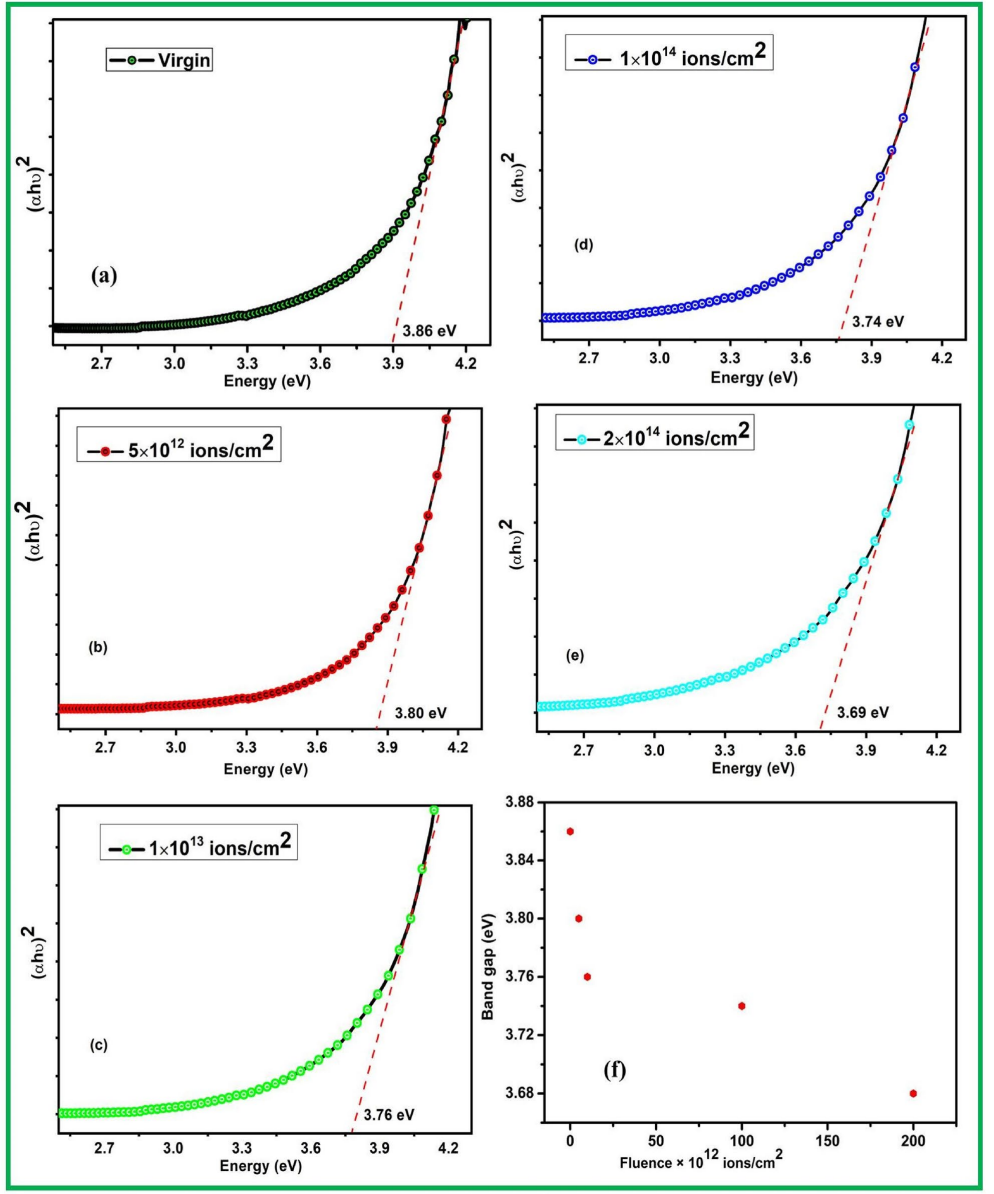
Ni^7+^ ion beam irradiated ZrO_2_ thin films shows the band edge of ZrO_2_ in all samples and Tauc’s plot with optical band gap energy of (**a**) Virgin, (**b**) 5 × 10^12^, (**c**) 1 × 10^13^ (**d**) 1 × 10^14^ and (**e**) 2 × 10^14^ ions/cm^2^ (**f**) plot of band gap values of virgin and irradiated samples.

Figure 6a–e shows the direct band of virgin and irradiated ZrO_2_ thin films that has been determined using the Tauc’s plot from the following equation:

3 $$\alpha {\text{h}}\upsilon = {\text{B}}\;\left( {{\text{h}}\upsilon - {\text{E}}_{{\text{g}}} } \right)^{{\text{r}}}$$

The values of optical band gap have been determined by extrapolation of linear fit from the absorption edge determining the intercept value on x- axis. The direct band gap of the virgin thin film is 3.86 eV and similar values also have been determined^49^. The band gap is little less than the bulk ZrO_2_ and reported in our previous work that may be due to quantum confinement in nanocrystalline ZrO_2_^60,61^. In case of irradiated samples, the values of band gap decrease with increasing in fluence in ZrO_2_ samples (band gap of 5 × 10^12^—3.80 eV, 1 × 10^13^—3.76 eV, 1 × 10^14^—3.74 eV and 2 × 10^14^—3.69 eV ions/cm^2^) as shown in Fig. 6f. In earlier reports, similar mitigation of optical band gap with increasing ions fluence has also been reported. This signifies that the valance band and conduction band are modified to various extent with ion fluence. Moreover, Ni ions beam irradiation produce point defects: vacancies, interstitials and lattice defects. The decrease in band gap with ions fluence might be attributed to effect of band tailing, owing to the defects produced during swift heavy ion beam irradiation^62^. During swift heavy ion irradiation, the velocity of incident ions is comparable or more than the velocity of electrons which leads in an inelastic scattering that cause track formation, phase transformation, annealing effects, modifications and amorphization etc. The sudden energy transfer results into the localized thermal hating of the samples and by the process of electron–phonon coupling, the energy is transferred to the sub atomic system^63^.

### Chemical properties of ZrO_2_ thin films

**Figure 7:**
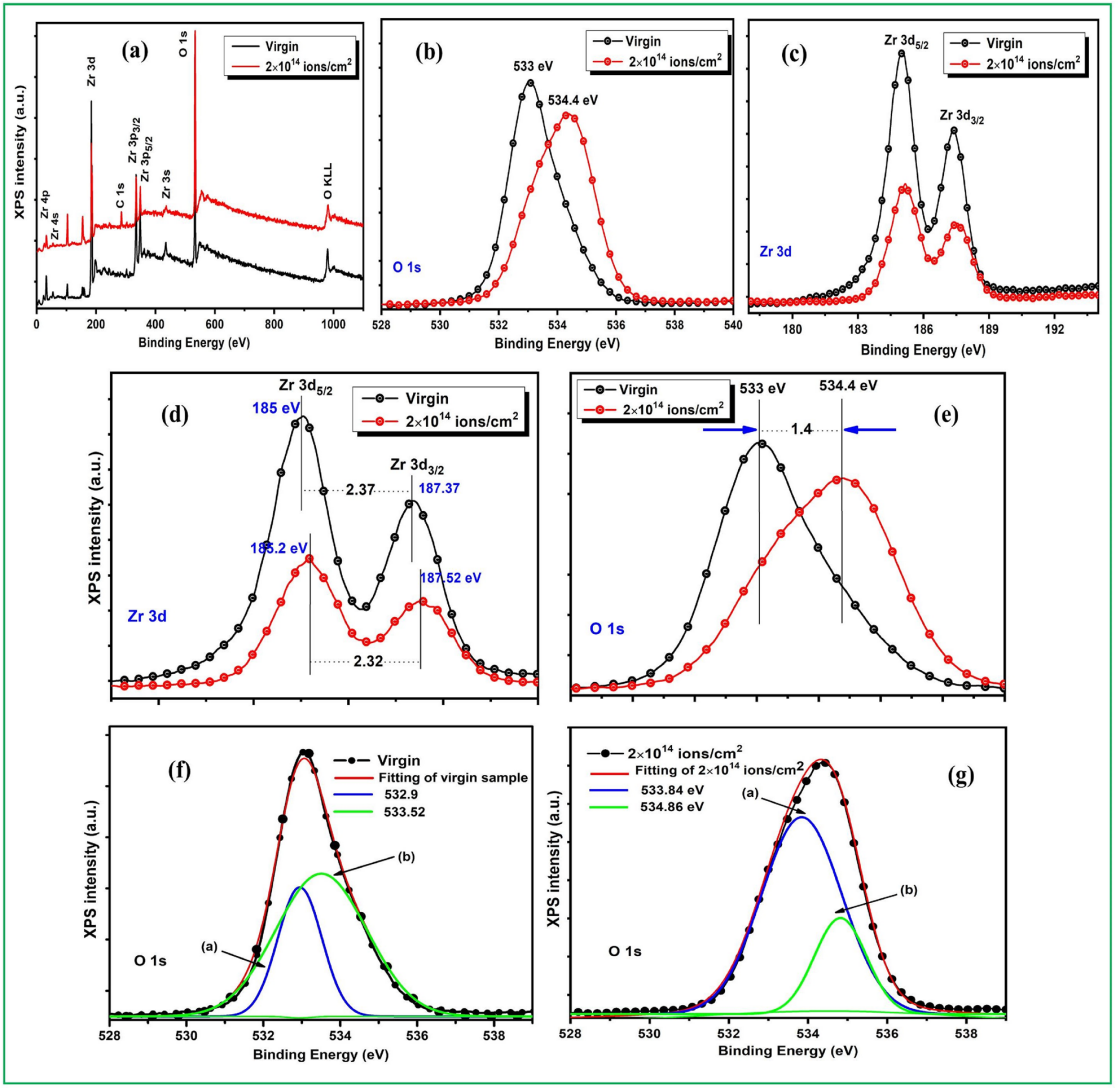
XPS spectra: (**a**) survey spectra of ZrO_2_ samples, (**b**) high resolution spectrum of O 1s (**c**) high resolution spectrum of Zr 3d of virgin and high fluence (2E14 ions/cm^2^) irradiated ZrO_2_ thin films, (**d**) Zr 3d determining the peak shifting and spin- orbit splitting (δ_3d_) (**e**) shifting of O 1s core level by 1.4 eV towards higher binding energy and curve fitting of O 1score level of (**f**) virgin (**g**) irradiated sample at higher fluence 2 × 10^14^ ions/cm^2^.

Figure 7a–c illustrate the survey spectra of Zr 3d and O 1s core energy levels respectively for the virgin and higher fluence (2 × 10^14^ ions/cm^2^) irradiated ZrO_2_ thin films measured using x-ray photoelectron spectroscopy (XPS). All photoelectron spectra were calibrated relative to the reference carbon C 1s peak at 285.0 eV^64^. From survey spectra, very strong Zr and O peak corresponds to Zr 3d and O 1s can be observed at binding energy of 184.41 eV and 533. 83 eV respectively. Other peaks for Zr 4p, Zr 4s, Zr 3p, Zr 3s and Auger O KLL are also observed at different binding energies as tabulated in Table 2 which are in agreement with the reported results^65^. The presence of carbon in the XPS spectra can be due to presence of adventitious carbon. Figure 7d,e showed the peak shifting of O 1s and Zr 3d core level towards higher binding energy, strong spin–orbit doublet of Zr 3d_5/2_-Zr 3d_3/2_ obtained with splitting (δ_3d_) 2.37 eV and 2.32 eV for virgin and irradiated samples respectively as tabulated in Table 3. The minor peak shifting (0.2 eV) of Zr 3d peak and significant peak shifting (1.4 eV) of O 1s spectra is observed as virgin sample is compared to the higher irradiated sample that might be due to the induced defects due to high energy irradiation^66^. The intensity of XPS peaks increased for the irradiated sample relative to the virgin sample. XPS of solid materials consist core—levels and valance band (VB) and the intensity of these levels increases with increase in binding energy. Particularly, the binding energy of core electrons is affected by local electron density and around the atom the arrangement (chemical bonding) for other atoms that generally leads to the chemical shifting in XPS spectra^67^. The chemical states of the ZrO_2_ thin films were analyzed gain an insight into the irradiation conditions dependence of the physical properties. The O 1s core level spectra of virgin and 2 × 10^14^ ions/cm^2^ were fitted by Gaussian fitting shown in Fig. 7f,g by solid line shapes. In O 1s fitted spectrum Fig. 7g, the binding energies for two components (a) and (b) corresponds to oxygen ion (O^2−^) combined with the metal cations in ZrO_2_ samples and oxygen ions located in oxygen—vacancy regions in structure and the bonding of the oxygen in form of O_2_, OH^−^ and H_2_O on the surface of the films^68^.The values of the obtained binding energy confirm the formation of ZrO_2_. The composition of ZrO_2_ thin films can be ascertained from the area under the peaks and relative sensitivity factor (RSF) of the different component. The value of RSF 1.45 and 0.57 for the peak Zr 3d and O 1s have been used to determine the atomic percentage of ZrO_2_ thin films^69^. The atomic percentage of Zr 3d and O 1s have been determined 33.5% and 66.8% respectively. XPS is more sensitive to estimate the ratio of species formed than Raman spectroscopy to measure the peak area percentages.

**Table 2:** Survey spectra of virgin and 2 × 10^14^ ions/cm^2^ determining the core level regions with their binding energies.

S. no	Core level regions	Binding energy (eV) of core level regions
1	Zr 4p	32.66
2	Zr 4s	54.79
3	Zr 3d	184.26
4	C 1s	285.41
5	Zr 3p3/2	334.95
6	Zr 3p1/2	349
7	Zr 3s	436.60
8	O 1s	533.64
9	O KLL	981.46

**Table 3:** Summary of the chemical properties of the films determining the binding energy of Zr 3d_5/2,_ Zr 3d_3/2_ and δ_3d_ (energy separation between the Zr 3d_5/2_ and Zr 3d_3/2_ levels) and O 1s using XPS.

S. no	Sample ions cm^−2^	Binding energy (eV)					
		Zr 3d_5/2_	Zr 3d_3/2_	Spin- orbit splitting (δ_3d_)	O 1s	O 1s (a)	O 1s (b)
1.	Virgin	185	187.37	2.37	533	532.9	534.86
2.	2 × 10^14^	185.2	187.52	2.32	534.4	533.84	534.86

### Surface morphology

**Figure 8:**
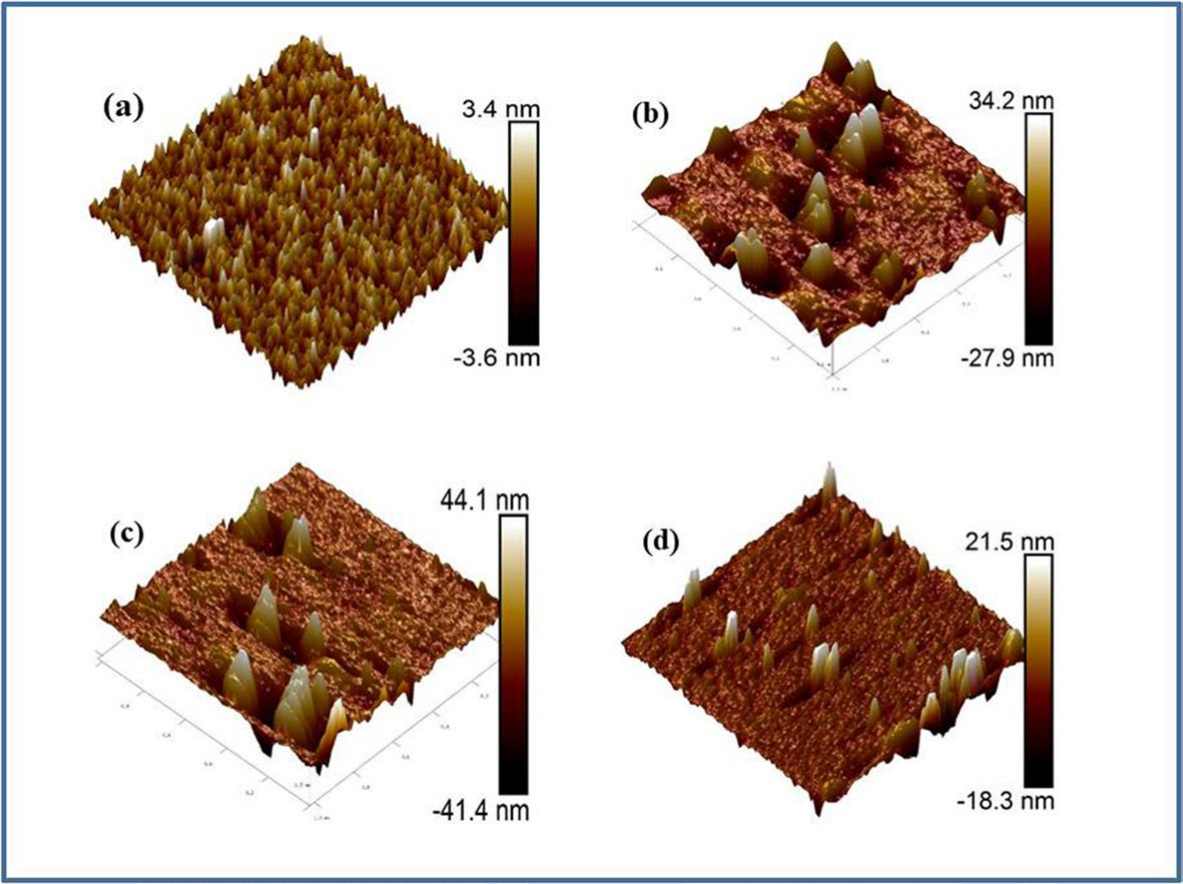
AFM micrograph of (**a**) virgin and 100 MeV 100 MeV Ni^7+^ ion beam irradiated ZrO_2_ thin films with varying fluence (**b**) 1 × 10^12^ ions/cm^2^ (**c**) 1 × 10^14^ ions/cm^2^ and (**d**) 2 × 10^14^ ions/cm^2^.

The surface morphology of virgin and 100 MeV ion irradiated ZrO_2_ thin films with the distinct ions fluence have been demonstrated by utilizing AFM (Atomic force microscopy) and associated micrographs are given in Fig. 8. Analysis of micrographs was analyzed at the surface top. AFM analysis was done at the distinct portion of thin films in tapping mode with the 20° tip cone angle and with the 10 nm of silicon probe tip radius. 2 × 2 µm lateral surface area has been considered by utilizing software of nanoscope analysis to determine the grain size, average roughness and RMS roughness of ZrO_2_ thin films. Average grain size of virgin and ion irradiated samples was demonstrated by utilizing Gaussian distribution. The average grain size of virgin thin films is found to be 35 nm.

After ion beam irradiation enhancement in grain size is observed i.e. 106.5 nm for the fluence 1 × 10^12^ ions/cm^2^. Grains distribution was remarked to be non-uniform on thin films surface after ion beam irradiation. For rest of the samples, the grain size might increase which is not possible to observe by the AFM. This increase in the grain size may be attributed to decrease in thermal energy of the substrate with increasing the fluence^23^. The enhancement in grain size is the result of smaller gains at specific sites of nucleation by energy imparted by incident ion beam. These consequences proposed that alterations at micro scale are less important in contrast to nano levels which are quite considerable. For the conception of alteration of material such as disorder in phase, amorphization, recrystallization by ion beam irradiation numerous models have been used. These models are named as lattice instability model^70^, thermal spike model^71,72^ and coulomb explosion model^73^. Average roughness (R_a_) increases (0.37 to 0.5 nm) with increasing ions fluence as compared to virgin sample. Also, the value of root mean square roughness (R_q_) increases from 0.52 nm to 0.66 nm with increasing fluence. The significant alterations in shape and size of the grains and R_q_ at elevated fluence is ascribed to the electronic excitations with high density instigated by SHI irradiation under the influence of numerous ion impact near the surface of thin films^74^.

## Conclusion

The 100 MeV Ni ion beam irradiation induced alterations in functional properties of RF grown ZrO_2_ thin films have been demonstrated in a successful manner. After 100 MeV Ni ion beam irradiation escalation, the XRD peaks are remarked and correlated with the reduction in originated defects density through the process of annihilation. Diffraction peaks correspond to the distinct crystal planes validates phase transformation from monoclinic to tetragonal structure of ZrO_2_. In SHI inelastic collisions takes place follows energy transfer which leads to heating and escalation in defects takes place which is responsible for variation in PL emission bands of ZrO_2_ thin films. SHI irradiation instigates the disorder in ZrO_2_ structure validates by Raman active modes and A_g_ and B_g_ Raman modes parallel to the monoclinic phase of ZrO_2_ is inferred. Significant reduction in band gap is remarked after enhancement in ion beam irradiation signifies that the conduction band and valence bands are modified by the ion beam irradiation. Significant shifting in ZrO_2_ core levels spectra towards higher binding energy is demonstrated in irradiated sample contrary to virgin sample on account of induction of defects due to high energy beam irradiation. Morphological analysis suggests enhancement in grain size and roughness, these modifications in size and shape of the grains with increasing Ni ion beam fluence is attributed to the electronic excitations caused by swift heavy ion beam irradiation under the effect of various ion impacts near the thin films surface. Due to origination of thermal spike, displacement spike, energy spike and distortion in lattice is originated after ion beam irradiation. This instigates the origination of defects and mutual annihilation of it responsible for alternations in functional properties ZrO_2_ thin films.
